# *PLTP* is a p53 target gene with roles in cancer growth suppression and ferroptosis

**DOI:** 10.1016/j.jbc.2022.102637

**Published:** 2022-10-26

**Authors:** Keerthana Gnanapradeepan, Alexandra Indeglia, David C. Stieg, Nicole Clarke, Chunlei Shao, James F. Dougherty, Nivitha Murali, Maureen E. Murphy

**Affiliations:** 1 Program in Molecular and Cellular Oncogenesis, The Wistar Institute, Philadelphia Pennsylvania, USA; 2 Graduate Group in Biochemistry and Molecular Biophysics, The University of Pennsylvania Perelman School of Medicine, Philadelphia, Pennsylvania, USA

**Keywords:** p53, tumor suppressor, lipid droplet, lipid peroxidation, liver cancer, PLTP, HDL, high density lipoprotein, LCL, lymphoblastoid cell line, MEF, mouse embryo fibroblast, PLTP, phospholipid transfer protein, qRT-PCR, quantitative RT-PCR, TBH, tert-Butyl-hydroperoxide

## Abstract

The tumor suppressor protein p53 suppresses cancer by regulating processes such as apoptosis, cell cycle arrest, senescence, and ferroptosis, which is an iron-mediated and lipid peroxide–induced cell death pathway. Whereas numerous p53 target genes have been identified, only a few appear to be critical for the suppression of tumor growth. Additionally, while ferroptosis is clearly implicated in tumor suppression by p53, few p53 target genes with roles in ferroptosis have been identified. We have previously studied germline missense p53 variants that are hypomorphic or display reduced activity. These hypomorphic variants are associated with increased risk for cancer, but they retain the majority of p53 transcriptional function; as such, study of the transcriptional targets of these hypomorphs has the potential to reveal the identity of other genes important for p53-mediated tumor suppression. Here, using RNA-seq in lymphoblastoid cell lines, we identify *PLTP* (phospholipid transfer protein) as a p53 target gene that shows impaired transactivation by three different cancer-associated p53 hypomorphs: P47S (Pro47Ser, rs1800371), Y107H (Tyr107His, rs368771578), and G334R (Gly334Arg, rs78378222). We show that enforced expression of *PLTP* potently suppresses colony formation in human tumor cell lines. We also demonstrate that PLTP regulates the sensitivity of cells to ferroptosis. Taken together, our findings reveal PLTP to be a p53 target gene that is extremely sensitive to p53 transcriptional function and which has roles in growth suppression and ferroptosis.

The *TP53* gene is the most frequently mutated gene in cancer and has a well-established role in tumor suppression. In order to suppress tumorigenesis, p53 acts as a sequence-specific transcription factor to regulate the transcription of several hundred target genes (1, 2). It is generally believed that the ability of p53 to induce target genes with roles in cell cycle arrest, senescence, and apoptosis is critical for tumor suppression by this protein. Along these lines, several p53 target genes with roles in senescence and apoptosis have been identified, including *CDKN1A*, *BAX*, *BBC3* (Puma), and *PMAIP1* (Noxa). However, there remain gaps in our understanding of p53. For example, the transactivation-deficient QS mutant of p53 (Leu25Trp26 to Gln25Ser26), which is unable to transactivate the majority of p53 target genes, retains tumor suppressor activity (3, 4). Triple KO mice for three key p53 target genes, *CDKN1A*, *BBC3*, and *PMAIP1*, do not show increased rates of spontaneous cancer (5). This has led to the perception that key p53 target genes with roles in tumor suppression remain to be identified. Along these lines, recent elegant CRISPR and RNAi screens have revealed p53 target genes with tumor suppressor activity; one of these is Zmat3, which plays a role in splicing (6). Additionally, analysis of an acetylation-deficient form of p53 called 3 KR pointed to the regulation of genes involved in ferroptosis as key for tumor suppression by p53 (7, 8).

Ferroptosis is an iron-mediated, caspase-independent form of cell death that is controlled by the peroxidation of polyunsaturated fatty acids. It is a process that differs from other forms of cell death on a genetic, biochemical, and morphological level (9). Key to ferroptosis is the enzyme GPX4, which functions in a glutathione-regulated manner to neutralize the harmful lipid reactive oxygen species that build up at the cellular membrane. Inhibition of GPX4 by the compound RSL3 or inhibition of the cystine importer SLC7A11 and the consequent decrease in glutathione levels caused by erastin lead to ferroptosis (10). Lipid metabolism plays a key role in ferroptosis sensitivity; for example, Acyl-CoA synthetase long-chain family member 4 (ACSL4) regulates ferroptosis by converting free fatty acids into fatty CoA esters that are required for ferroptosis (11, 12). It is accepted that p53 regulates ferroptosis sensitivity; however, whether p53 plays a negative or a positive role has been somewhat controversial. Some groups have shown that p53 positively regulates the sensitivity of cells to ferroptosis, for example, by transcriptionally regulating critical genes in ferroptosis such as *SLC7A11* (8), *ALOX12* (13), *ALOX1*5 and *SAT1* (14), *MDM2* (15), and *GLS2* (16, 17). Other researchers have found that p53 can negatively regulate ferroptosis, for example, through induction of *CDKN1A/p21* (18) or by blocking dipeptidyl-peptidase-4 activity (19). Whether p53 positively or negatively regulates the sensitivity of cells to ferroptosis appears to be cell-type– and stimulus-specific.

Our lab previously generated a mouse model for a single nucleotide polymorphism in *TP53* that exists in approximately 1% of African-descent individuals and which encodes serine instead of proline at amino acid 47 of p53 (Pro47Ser or rs1800371; hereafter P47S). The P47S variant of p53 is impaired for phosphorylation on serine 46 (20), and the P47S mouse is highly tumor prone, particularly to hepatocellular carcinoma. We discovered that the P47S variant is defective in the regulation of ferroptosis, in part due to impaired ability to regulate the p53 target genes *GLS2* and *SLC7A11* (17). We also reported that P47S cells show increased synthesis of anti-oxidants like coenzyme A and glutathione and that this contributes to the ferroptotic defect (21, 22). Additionally, we found that the P47S variant is associated with increased risk for premenopausal breast cancer in African-descent women (23). Finally, we reported that the ferroptotic defect in humans and mice with the P47S variant leads to increased iron accumulation in cells and that the P47S variant is significantly associated with markers of Iron Overload disorder in African-descent individuals (24). Our combined data suggest that the P47S variant of p53 is hypomorphic in function; that is, this variant transactivates the majority of p53 target genes and is proficient at inducing growth arrest, senescence, and apoptosis. However, P47S is defective for ferroptosis and tumor suppression. We reasoned that identifying p53 target genes that show impaired transactivation in P47S cells had the potential to reveal genes with roles in ferroptosis and tumor suppression. Here, we identify a p53 target gene, *PLTP* (phospholipid transfer protein), that shows impaired induction in P47S cells, as well as in cells containing two other hypomorphic variants of p53, Y107H, and G334R. These data suggest that the transactivation of *PLTP* may be extremely sensitive to the level of functional p53 in the cell.

The *PLTP* gene encodes a ubiquitously expressed lipid transfer protein that exists as a monomeric protein of approximately 80 kDa in size, though isoforms of both larger and smaller sizes are reported (25). PLTP belongs to a family of lipid transfer/lipopolysaccharide-binding proteins that include cholesterol ester transfer protein and lipopolysaccharide-binding protein. Besides transferring phospholipids, PLTP also transfers diacylglycerol, α-tocopherol, cerebroside, and lipopolysaccharides (26). As such, PLTP is typically viewed as a relatively nonspecific lipid transfer protein. Perhaps, the best characterized activity of PLTP is the transfer of phospholipids from triglyceride-rich lipoproteins to high density lipoprotein (HDL), thus regulating the size of HDL particles. In addition, PLTP is believed to be involved in cholesterol metabolism. In mouse models, PLTP overexpression induces atherosclerosis, while its deficiency reduces it (25). PLTP KO mice show a complete loss of phosphatidylcholine, phosphatidylethanolamine, phosphatidylinositol, and sphingomyelin, along with a partial loss of free cholesterol activity (27). We show that when exogenously overexpressed, PLTP is a potent suppressor of colony formation in cancer cells and that it plays a role in controlling the sensitivity of cells to ferroptosis. Our data suggest that PLTP plays a role in lipid droplet accumulation and that lipid droplet formation may influence the sensitivity of cells to ferroptosis.

## Results

### PLTP shows decreased transactivation by the P47S variant of p53

**Figure 1:**
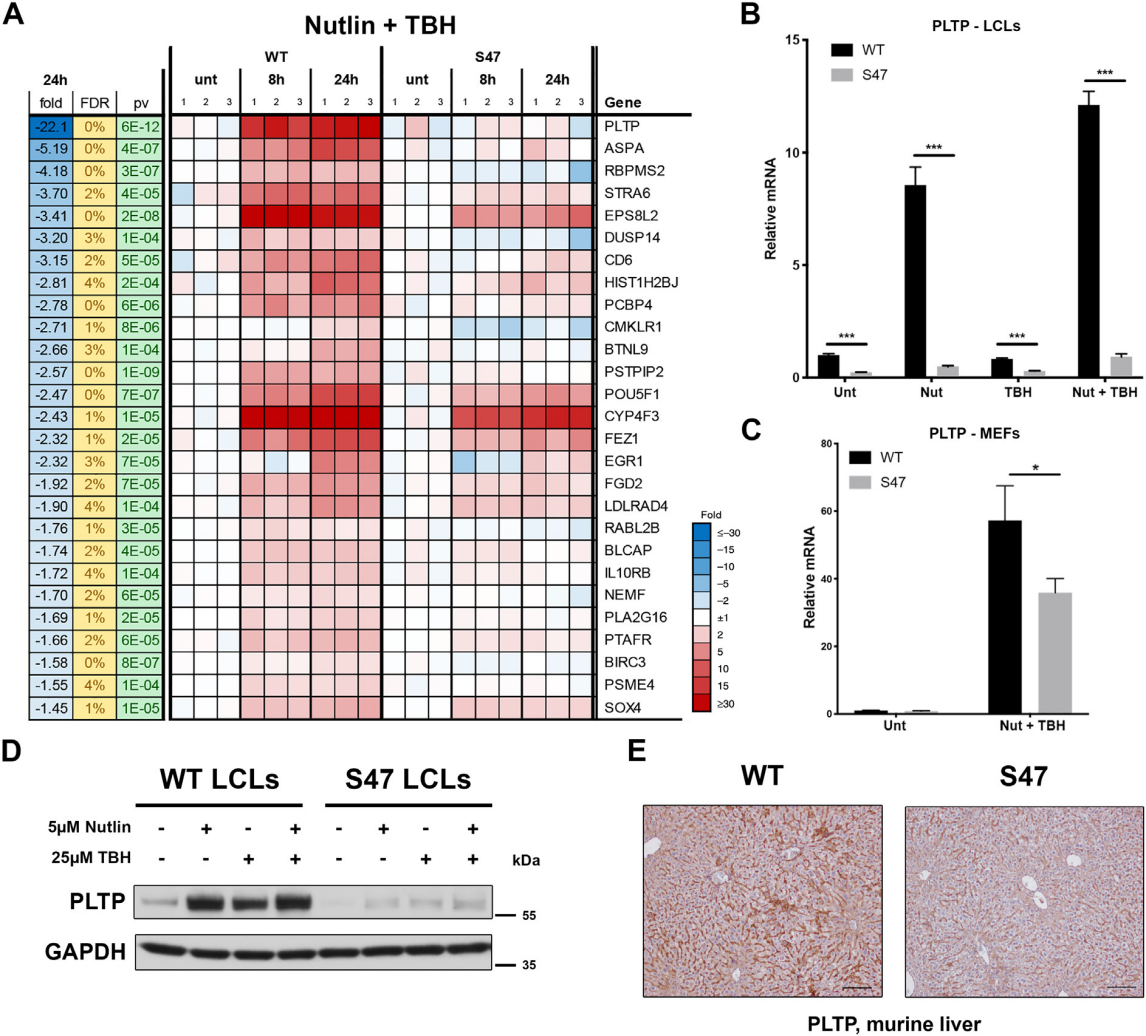
**PLTP is differentially regulated by WT p53 and the P47S variant**. *A*, heat map from RNA-Seq data depicting significantly altered genes in WT and P47S LCLs treated with 5 μM nutlin and 25 μM TBH for 0, 8, and 24 h, performed in triplicate. Genes showing 1.5 fold or greater increased induction in WT compared to P47S with false discovery rate under 5% are shown. *B*, qRT-PCR analysis of *PLTP* in LCLs treated with 5 μM nutlin, 25 μM TBH, or combination of both, for 24 h. All values were normalized to a control gene (18S); n = 3, error bars indicate SD. (∗∗∗) *p*-value < 0.001, Student’s *t* test. *C*, qRT-PCR analysis of *Pltp* in MEFs treated with 10 μM nutlin and 25 μM TBH for 24 h. All values were normalized to a control gene (Cyclophilin A); n = 3, error bars indicate SD. (∗) *p*-value < 0.05, Student’s *t* test. *D*, Western blot analyses of PLTP expression in WT and P47S LCLs treated with 5 μM nutlin, 25 μM TBH, or combination of both, for 24 h. *E*, immunohistochemical analysis of PLTP protein in WT and P47S liver tissue. Data are representative of n = 4 liver sections per genotype. Scale bar represents 100 μm. LCL, lymphoblastoid cell line; MEF, mouse embryo fibroblast; PLTP, phospholipid transfer protein; qRT-PCR, quantitative RT-PCR; TBH, tert-Butyl-hydroperoxide.

Previously, we showed that mice containing the P47S variant of p53 are highly tumor-prone and that cells and tissues from these mice are resistant to ferroptosis (17). Here, we sought to use P47S as a tool to identify other p53 target genes that might play roles in tumor suppression and ferroptosis. Toward this goal, we employed immortalized human lymphoblastoid cell lines (LCLs) from two individuals from the same region of Africa that are homozygous for WT p53 or the P47S variant. These cell lines were treated with nutlin, which stabilizes p53, combined with tert-Butyl-hydroperoxide (TBH); this combination has been shown to induce ferroptosis in a p53-regulated manner (14). RNA-seq of these samples revealed *PLTP* as the top gene that was induced in WT LCLs but not P47S (>20-fold difference), out of approximately two dozen genes that were impaired for upregulation in P47S (Fig. 1*A*). Of these two dozen P47S-impaired targets, the majority have been identified as p53 targets in multiple studies (http://www.targetgenereg.org/), and none were identified previously in a small, targeted QPCR study of P47S cells (17). All other classical p53 targets, including *CDKN1A*, *BBC3*, and *PMAIP1*, were transactivated identically in WT and P47S cells.

*PLTP* encodes a phospholipid transfer protein, and this gene is a known direct p53 target gene (28). Quantitative RT-PCR (qRT-PCR) in LCLs confirmed that *PLTP* was significantly induced by nutlin or the nutlin/TBH combination in WT but not P47S LCLs (Fig. 1*B*). We next analyzed the induction of *PLTP* in mouse embryo fibroblasts (MEFs) from mice containing WT p53 or the P47S variant. We again found that *Pltp* shows decreased transactivation in P47S MEFs compared to WT, though there was clearly some transactivation of *Pltp* in P47S MEFs (Fig. 1*C*). Western blot analyses confirmed that the protein levels of PLTP are significantly decreased in P47S LCLs (Fig. 1*D*), and immunohistochemistry revealed decreased PLTP protein in P47S mouse livers, compared to age- and sex-matched WT controls (Fig. 1*E*). These collective data demonstrate that PLTP is expressed at lower levels in human and mouse P47S cell lines, as well as in tissues from P47S mice.

### PLTP shows decreased transactivation by multiple p53 hypomorphs, along with potent colony suppressive activity

**Figure 2:**
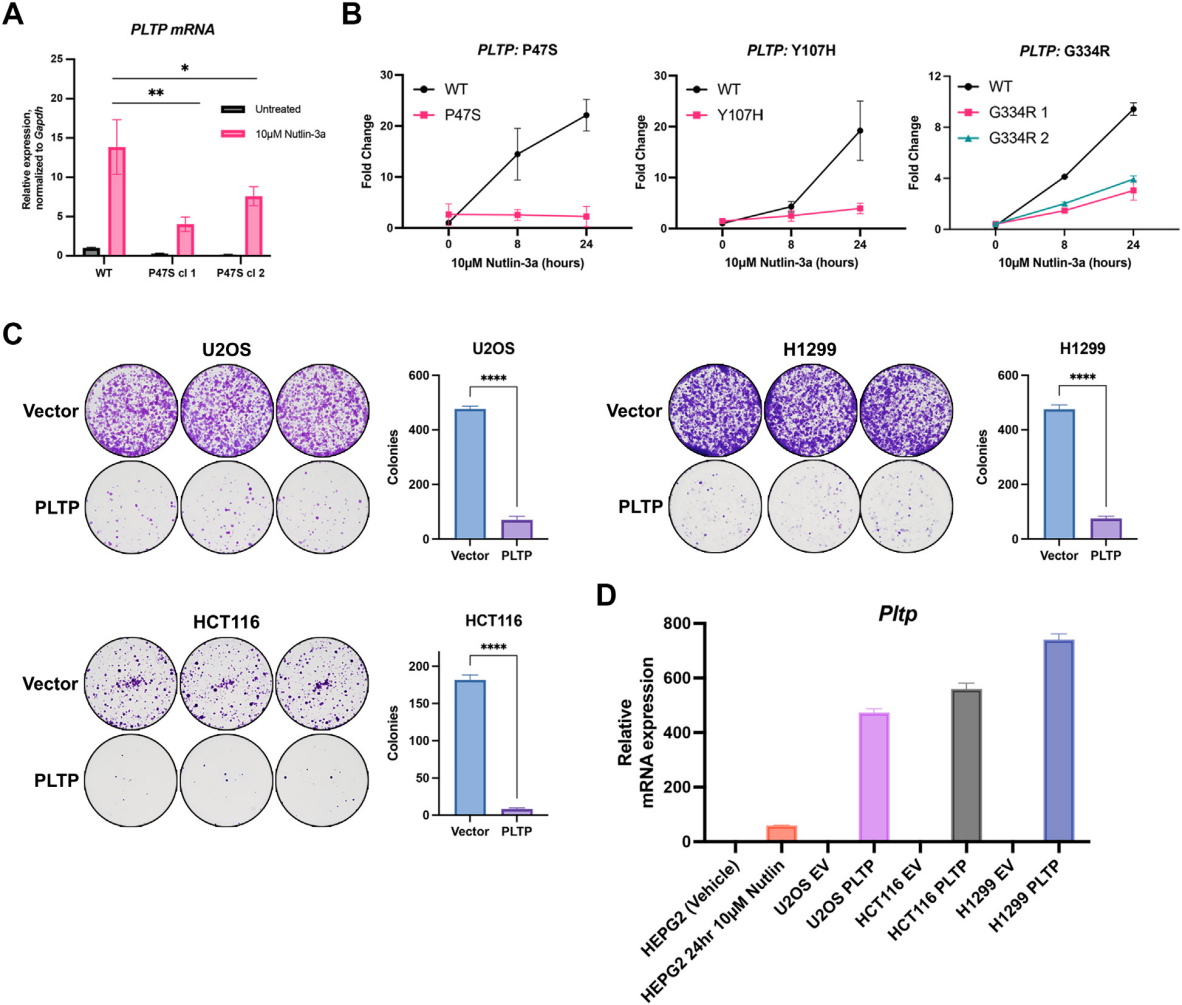
**Impaired induction of PLTP in multiple p53 hypomorph cell lines; significant colony suppressive ability of PLTP**. *A*, qRT-PCR analysis of *PLTP* in HepG2 cells with WT p53 and in two independent P47S clones generated by CRISPR knock-in. All values were normalized to a control gene (*Gapdh*); n = 3, error bars indicate SD. (∗) *p*-value < 0.05; (∗∗) *p*-value 0.01, Student’s *t* test. *B*, fold RNA changes of *PLTP* mRNA, normalized to control, from RNA isolated from LCLs from three different p53 hypomorphs (P47S, Y107H, and G334R) compared to control LCLs from family members with WT p53, treated with 10 μM nutlin for 0, 8, and 24 h. Error bar indicates SD from three biological replicates. *C*, colony suppression assays of the cell lines indicated following transfection with vector control (pcDNA3.1) or pcDNA3.1-PLTP. Colonies were stained with crystal violet and counted using ImageJ. Shown are representative images of triplicate wells along with quantification, including SD. (∗∗∗∗) *p*-value 0.0001, Students *t* test. *D*, qRT-PCR analysis of *Pltp* in transfected cell lines in (*C*), compared to *Pltp* in HEPG2 cells following 10 μM nutlin treatment for 24 h. All values were normalized to a control gene (*Gapdh*); n = 3, error bars indicate SD. LCL, lymphoblastoid cell line; PLTP, phospholipid transfer protein; qRT-PCR, quantitative RT-PCR.

To test the impact of the P47S variant in another cell background, we next created two independent clones of HepG2 cells in which we used CRISPR/Cas9 to convert WT p53 to the homozygous P47S variant; DNA sequencing confirmed no other mutations in p53 (see Materials and Methods). Treatment with nutlin followed by qRT-PCR revealed that *PLTP* mRNA is induced in parental HepG2 cells as previously described (28) but that this induction is significantly impaired in both P47S clones (Fig. 2*A*). We previously described another cancer-associated hypomorph of p53 called G334R. Like P47S, we found that G334R cells were also impaired for transactivation of *PLTP*, compared to LCLs from family members with WT p53 (29). These data raised the possibility that transactivation of PLTP might be extremely sensitive to the function of p53, perhaps even a hallmark of hypomorphic p53. To address this question, we compared *PLTP* transactivation in nutlin-treated LCLs from individuals containing three different cancer-associated p53 hypomorphs: P47S, Y107H, and G334R. Like P47S, Y107H and G334R are both associated with increased cancer risk (29, 30). In each case, these cells were compared to ethnicity/region-matched LCLs containing WT p53. RNA analyses revealed that *PLTP* induction by nutlin was significantly decreased in LCLs from P47S, Y107H, and G334R individuals compared to matched WT controls (Fig. 2*B*). To explore the possibility that PLTP might contribute to tumor suppression by p53, we employed colony suppression assays. These studies indicated that PLTP is a potent suppressor of colony formation in multiple cancer cell lines containing WT p53 (U2OS, Hct116) and in a p53-null cell line (H1299) (Fig. 2*C*). When we assessed the level of PLTP overexpression in these transiently transfected cells, we found 10-fold increased PLTP, compared to HepG2 cells treated with nutlin (Fig. 2*D*). This level of overexpression is typical in a transient transfection/colony suppression assay, and these data support the premise that PLTP can be growth suppressive. That said, we cannot exclude the possibility that PLTP is growth suppressive only when expressed at extremely high levels.

### PLTP regulates ferroptosis sensitivity

**Figure 3:**
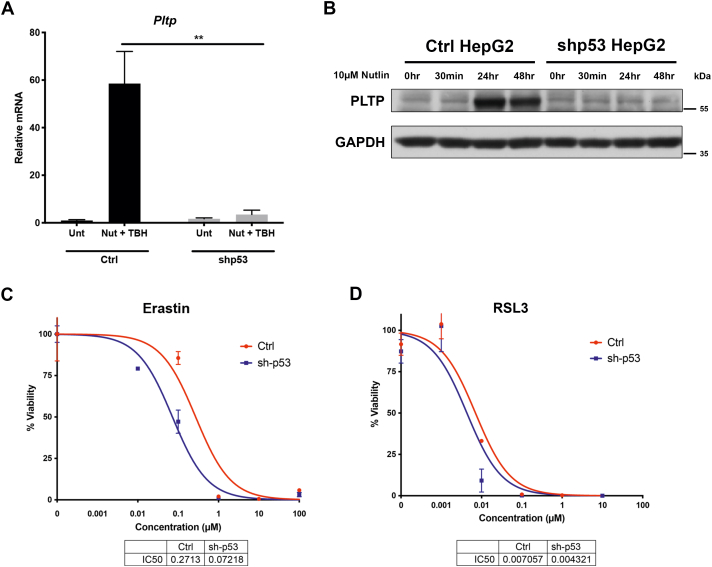
**p53 positively regulates PLTP but negatively regulates ferroptosis in HepG2 cells**. *A*, qRT-PCR analysis of *PLTP* in HepG2 cells treated with 10 μM nutlin for 24 h, with and without p53 short hairpin (shp53). All values were normalized to a control gene (18S); n = 3, error bars indicate SD. (∗∗) *p*-value < 0.01, Student’s *t* test. *B*, Western blot analyses comparing PLTP expression in HepG2 cells with and without p53 knockdown. Cells were treated with 10 μM nutlin and lysates were harvested at 0 h, 30 min, 24 h, and 48 h. Data are representative of three independent experiments. *C* and *D*, viability analysis of HepG2 cells with and without p53 knockdown treated with indicated doses of (*C*) erastin or (*D*) RSL3 for 72 h. Error bars represent SEM, n = 4 biological replicates. PLTP, phospholipid transfer protein; qRT-PCR, quantitative RT-PCR.

**Figure 4:**
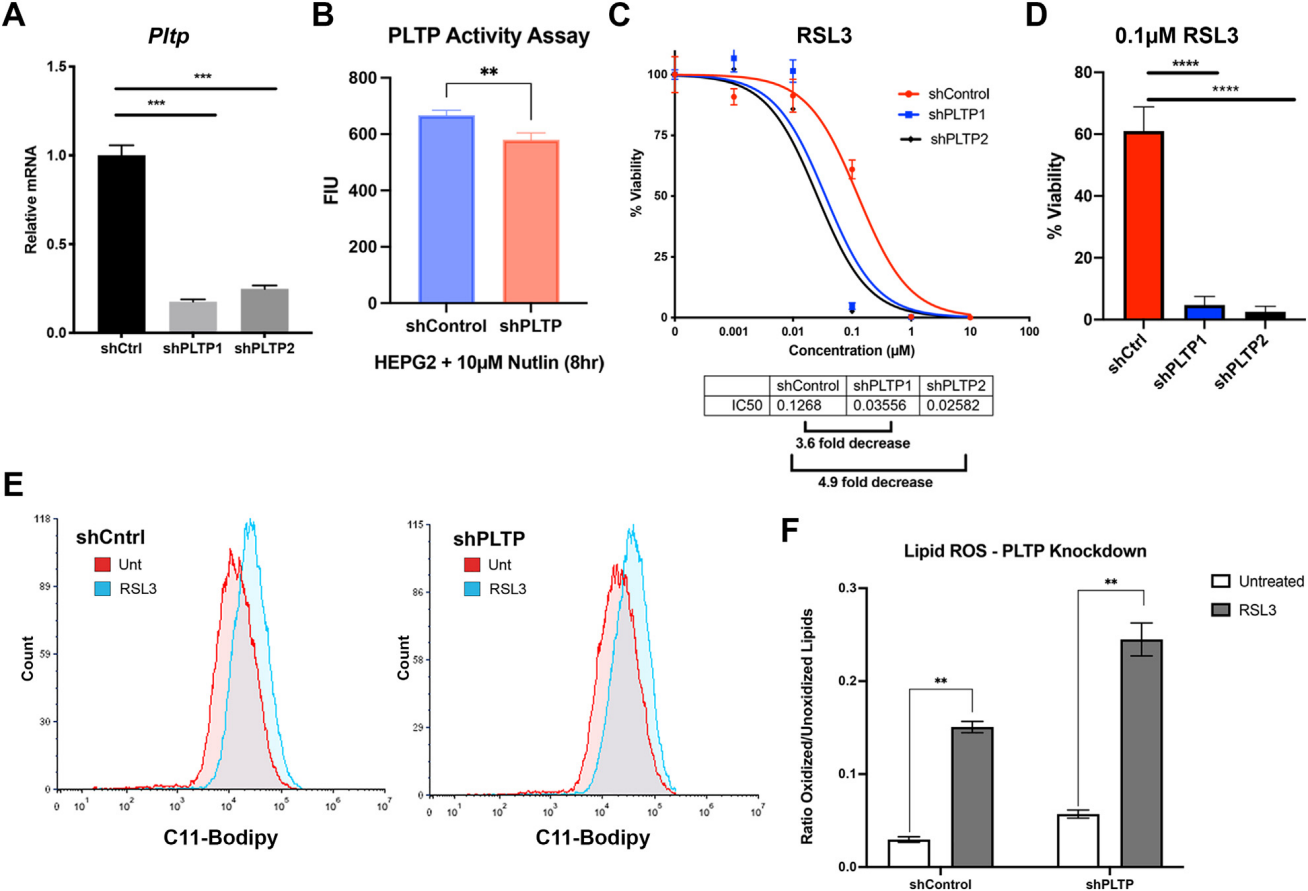
**Knockdown of PLTP increases sensitivity to RSL3-induced ferroptosis**. *A*, qRT-PCR analysis of *PLTP* expression in the indicated *PLTP* knockdown HepG2 cells. All values were normalized to a control gene (18S); n = 3, error bars indicate SD. (∗∗∗) *p*-value < 0.001, Student’s *t* test. *B*, activity of PLTP in shControl and shPLTP HEPG2 cells following treatment with 10 μM Nutlin for 8 h. Shown by mean fluorescence intensity units (FIU). n = 3, error bars indicate SD. (∗∗) *p*-value < 0.01, Student’s *t* test. *C*, viability analysis of short hairpin control or *PLTP* knockdown cells treated with indicated doses of RSL3 for 72 h. Data are representative of three independent experiments and four technical replicates; error bars represent SEM. *D*, viability of short hairpin control or PLTP knockdown cells treated 0.1 μM RSL3 for 72 h; viability was assessed using Alamar Blue assay. Error bars represent SEM, (∗∗∗∗) *p*-value < 0.0001, Student’s *t* test. *E*, lipid peroxidation in shControl or shPLTP knockdown cell lines treated with 0.5 μM RSL3 for 3 h, as assessed by flow cytometry using C11-BODIPY. Data are representative of three independent experiments. *F*, quantification of lipid peroxidation levels as ratio of oxidized/unoxidized signal. Error bars indicate SD. n = 3, (∗∗) *p*-value < 0.01. PLTP, phospholipid transfer protein; qRT-PCR, quantitative RT-PCR.

Given the role of lipids in ferroptosis and the role of PLTP in lipid transfer, we next sought to determine whether *PLTP* played a role in ferroptosis. We first wanted to determine the role of p53 in the sensitivity in HepG2 cells to ferroptosis; this was particularly important given the literature that p53 can positively or negatively regulate ferroptosis, depending on cell type and cell stress. To this end, we silenced p53 using a stably-transfected short hairpin, and we confirmed that the induction of *PLTP* by nutlin occurs in a p53-dependent manner (Fig. 3, *A* and *B*), thus confirming a previous report (28). We next found that both erastin and RSL3 efficiently induced ferroptosis in HepG2 cells and that silencing p53 led to a 3.8-fold increased sensitivity to erastin (Fig. 3*C*) but modest to no change in IC_50_ for RSL3 (Fig. 3*D*). These data suggested that p53 negatively regulates ferroptosis in HepG2 cells and further that PLTP might potentially confer resistance. To test the latter hypothesis, knockdown cell lines for *PLTP* were generated *via* stable expression of two different short hairpins for this gene in HepG2 cells; qRT-PCR confirmed that *PLTP* was effectively silenced (Fig. 4*A*). Not surprisingly, we found that PLTP enzymatic activity was modestly but significantly decreased in these knockdown cells (Fig. 4*B*). Knockdown of *PLTP* using these two different short hairpins led to a >3-fold increased sensitivity to ferroptosis induced by erastin (data not shown) or RSL3 (Fig. 4*C*); the latter was accompanied by a significant decrease in viability following treatment with 0.1 μM RSL3 (Fig. 4*D*). To confirm that the cell death observed following RSL3 treatment was due to ferroptosis, we used the ferroptosis inhibitors ferrostatin and liproxstatin. PLTP-silenced cells demonstrated nearly 100% cell death when treated with a high dose (1 μM) of RSL3. However, when these cells were pretreated for 30 min with either ferrostatin or liproxstatin, cell death was rescued (Fig. S1*A*). To extend these findings further, we examined the impact of PLTP silencing on the generation of lipid reactive oxygen species, which are a hallmark of ferroptosis. Consistent with our viability data, we found that silencing PLTP significantly increased the lipid peroxidation that occurs after treatment with RSL3 (Fig. 4, *E* and *F*).

**Figure 5:**
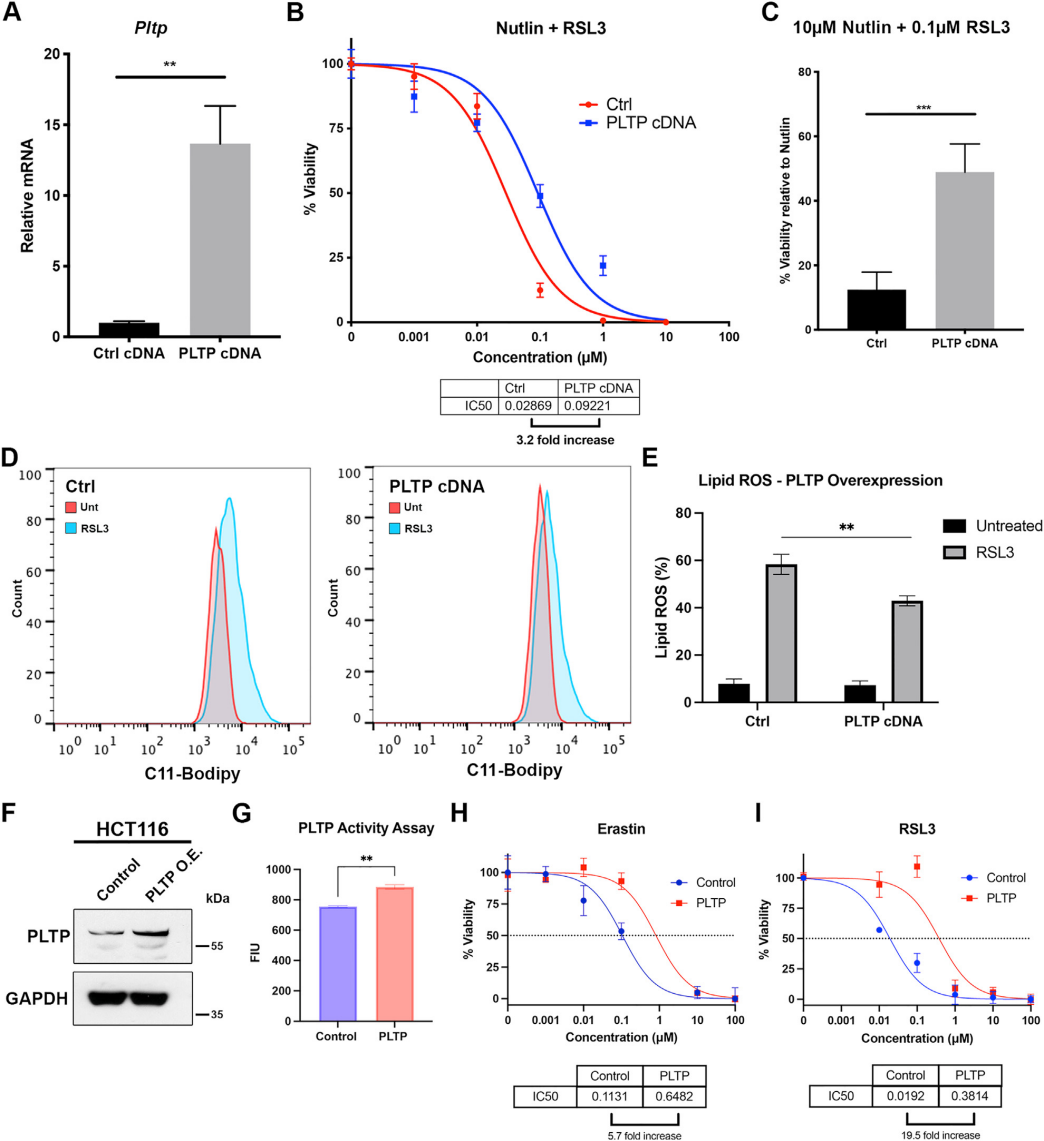
**Overexpression of PLTP confers ferroptosis resistance**. *A*, qRT-PCR analysis of *PLTP* expression HepG2 cells containing empty vector and PLTP cDNA. All values were normalized to a control gene (18S); n = 3, error bars indicate SD. (∗∗∗) *p*-value < 0.001, Student’s *t* test. *B*, viability analysis of vector control or cells overexpressing PLTP treated with 10 μM nutlin and the indicated doses of RSL3 for 72 h. Viability was normalized to treatment with 10 μM nutlin alone. Data are representative of three independent experiments and four technical replicates; error bars represent SEM. *C*, viability analysis of vector control or cells overexpressing PLTP treated with 10 μM nutlin and 0.1 μM RSL3 for 72 h. Error bars indicate SD, (∗∗∗) *p*-value < 0.001. *D*, lipid peroxidation in cells overexpressing PLTP treated with 0.5 μM RSL3 for 3 h, as assessed by flow cytometry using C11-BODIPY. Data are representative of three independent experiments. *E*, quantification of lipid peroxidation levels. Error bars indicate SD. n = 3, (∗∗) *p*-value < 0.01. *F*, Western blot analysis of *PLTP* expression HCT116 cells containing empty vector and PLTP cDNA. GAPDH used as a loading control. *G*, activity of PLTP in HCT116 cells containing empty vector and PLTP cDNA. Shown by mean fluorescence intensity units (FIU). n = 3, error bars indicate SD. (∗∗) *p*-value < 0.01, Student’s *t* test. *H* and *I*, viability analysis of vector control or cells overexpressing PLTP treated with the indicated doses of erastin (*H*) or RSL3 (*I*) for 72 h. Viability was normalized to vehicle control. Data are representative of four independent experiments technical replicates; error bars represent SD. PLTP, phospholipid transfer protein; qRT-PCR, quantitative RT-PCR.

We next sought to determine whether overexpression of PLTP conferred ferroptosis resistance in HepG2 cells by stably expressing a human PLTP expression construct in these cells. Overexpression of PLTP RNA was confirmed in pooled, stably-transfected cells (Fig. 5*A*). We next assessed cell viability following ferroptosis induction with RSL3. We found that PLTP overexpression led to increased resistance to RSL3-induced ferroptosis (Fig. 5*B*), as well as increased viability following treatment with nutlin plus RSL3 (Fig. 5*C*). Consistent with these data, we found that overexpression of PLTP also led to decreased lipid peroxidation induced by RSL3 (Fig. 5, *D* and *E*). We next tested this in an independent cell line, Hct116, and found that stable overexpression of PLTP led to increased PLTP protein level (Fig. 5*F*), enzymatic activity (Fig. 5*G*), and resistance to erastin and RSL3 (Fig. 5, *H* and *I*). Finally, we sought to determine whether PLTP might have a more broadly cytoprotective role, by assessing the IC_50_ of shControl and shPLTP HepG2 to cells to numerous other genotoxic and cytotoxic compounds. No significant differences in cell death were observed in PLTP-silenced cells when treated with cisplatin (Fig. S1, *B* and *C*). Similarly, we found no difference in the response of PLTP-silenced cells to other stressors, including doxorubicin, etoposide, camptothecin, and tunicamycin (Fig. S1, *D*–*G*). These data indicate that the role of PLTP in the resistance to cell death appears to be specific to ferroptosis. However, the mechanism underlying this contribution to ferroptosis was unclear.

### PLTP promotes lipid droplet formation

**Figure 6:**
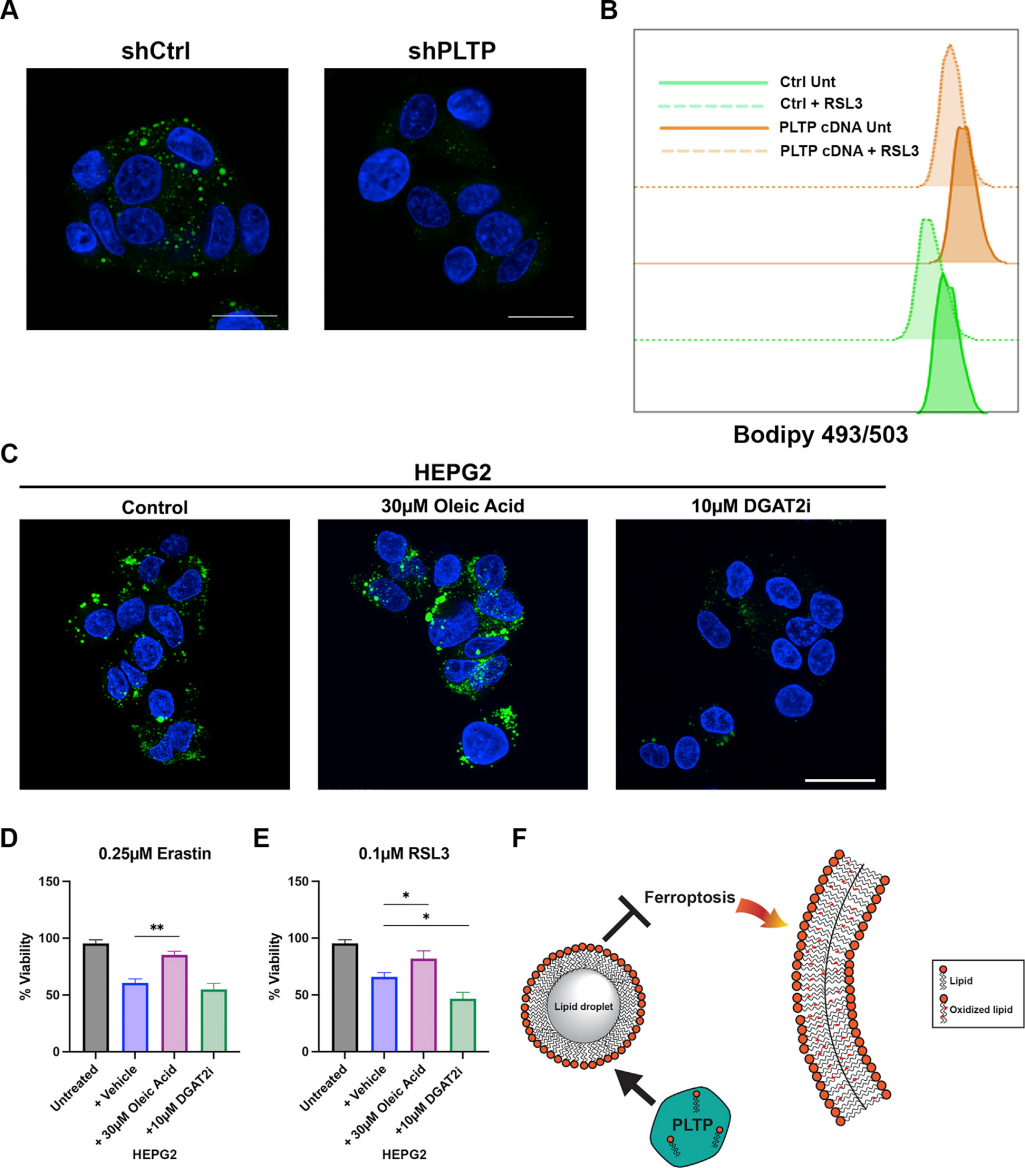
**PLTP controls lipid droplet content**. *A*, knockdown of *PLTP* decreases lipid droplet accumulation, visualized using BODIPY 493/503. DAPI staining was used to visualize nuclei, scale bar represents 20 μm. *B*, lipid droplet accumulation assessed using BODIPY 493/503 in cells overexpressing *PLTP*, treated with and without 500 nM RSL3 for 12 h. Data are representative of two independent experiments and four technical replicates of each. *C*, immunofluorescence of lipid droplet accumulation, visualized using BODIPY 493/503. HEPG2 cells were treated with vehicle control, 30 μM oleic acid, or 10 μM DGAT2i overnight to perturb lipid droplet accumulation. DAPI staining was used to visualize nuclei. Scale bar represents 25 μm. *D* and *E*, viability analysis of HEPG2 cells pretreated overnight with 30 μM oleic acid or 10 μM DGAT2i and then treated with the indicated doses of erastin (H) or RSL3 (I) for 72 h. Viability was normalized to untreated vehicle. Data are representative of three independent experiments technical replicates; error bars represent SD. (∗) *p*-value < 0.05, (∗∗) *p*-value < 0.01, Student’s *t* test. *F*, proposed mechanism for the role of PLTP in ferroptosis resistance: PLTP overexpression causes increased lipid droplet formation, thus preventing oxidized polyunsaturated fatty acids from accumulating in the plasma membrane. PLTP, phospholipid transfer protein.

We next sought to explore the potential mechanism whereby PLTP regulates ferroptosis resistance. PLTP is known to play a role in transporting lipids, including phosphatidylethanolamine; phosphatidylethanolamine is one of the key lipids that undergo peroxidation at the cell membrane during ferroptosis (31, 32). Along these lines, a recent study demonstrated that enhanced lipid storage, in the form of lipid droplets, can inhibit ferroptosis by sequestering polyunsaturated fatty acids away from the cell membrane (33, 34). Based on these findings, we decided to test whether PLTP might regulate the formation of lipid droplets. Toward this goal, we first examined the impact of PLTP silencing on lipid droplet formation using the BODIPY 493/503 marker. To corroborate these data, we used flow cytometry to measure lipid droplet formation following RSL3 treatment in cells stably-transfected with PLTP or control vector. Immunofluorescence using BODIPY 493/503 revealed readily observable lipid droplets in HepG2 cells stably-transfected with shControl; these were largely undetectable in shPLTP-expressing cells (Fig. 6*A*). Flow cytometry analysis of cells overexpressing PLTP showed an increase in lipid droplet content, as detected by the rightward shift of the solid orange curve (PLTP overexpressing) compared to the solid green curve (vector control) (Fig. 6*B*). Treatment with RSL3 led to a decrease in lipid droplet content in both cell lines, as shown by the leftward shift of the curves from the solid colors to the respective dotted colors (Fig. 6*B*). These latter data are consistent with previously reported data showing induction of ferroptosis leads to a decrease in lipid droplet levels (33). Notably, whereas RSL3 causes decreased lipid droplet accumulation in cells overexpressing PLTP, the overall lipid droplet levels remain higher in PLTP-overexpressing cells than control cells (Fig. 6*B*). To further support the possibility that lipid droplets may contribute to ferroptosis sensitivity, we treated HepG2 cells with vehicle, oleic acid (increases lipid droplets), or an inhibitor to DGAT2, which would block lipid droplet formation (35). Notably, oleic acid enhanced lipid droplets and protected cells from cell death by erastin and RSL3, while the DGAT inhibitor reduced lipid droplets and increased cell death by Rsl3 (Fig. 6, *C*–*E*). The combined data suggest that PLTP may promote ferroptosis resistance by enhancing the sequestration of lipids in lipid droplets and away from the plasma membrane (see model, Fig. 6*F*).

## Discussion

PLTP was previously identified in HepG2 cells as a direct p53 target gene with a role in lipid metabolism (28, 36). While this protein is reported to be widely expressed in many cell types, we were unable to detect PLTP following nutlin treatment in several tumor cell lines, with the exception of HepG2. Therefore, we selected HepG2 cells for our functional studies. Interestingly, we noted that PLTP overexpression caused significant growth inhibition in H1299 cells, which lack p53, suggesting that PLTP can be growth suppressive independent of WT p53. It remains unclear why PLTP is potently growth suppressive, while at the same time promoting ferroptosis resistance, as ferroptosis is widely accepted to lead to cell death. Because PLTP functions in lipid import, it is possible that low levels of PLTP promotes lipid droplets that protect cells from ferroptosis but that high levels of PLTP may promote lipid import to levels that are conducive to ferroptosis; this remains to be determined. Additionally, the type of lipid to be imported could dictate whether PLTP plays a role in the activation or inhibition of ferroptosis. For example, PLTP can promote the import of phosphatidylethanolamine, which would promote ferroptosis, or the import of α-tocopherol, which is a lipophilic antioxidant that protects cells from ferroptosis (37, 38). As such, the role of PLTP in ferroptosis may be, like p53, context dependent.

Insight into the function of PLTP comes from studies of the PLTP KO mouse. These mice lose the ability to transfer lipids such as phosphatidylcholine, phosphatidylethanolamine, phosphatidylinositol, and sphingomyelin, and they partially lose the ability to transfer-free cholesterol (27). These PLTP KO mice also have significantly lower levels of HDL cholesterol found in the plasma, due to impaired cholesterol absorption in the intestine (39), and they show decreased atherosclerosis (40). Finally, PLTP has been shown to regulate the phagocytic activity of macrophages and microglial cells, and PLTP-deficient mice express much lower levels of cytokine interleukin 6 and lower levels of infiltrating macrophages after stress (41). These combined studies suggest PLTP plays a complex role in mediating several processes ranging from lipid metabolism to immune function. It is tempting to speculate that the impaired regulation of PLTP in the P47S mouse could explain some of the defects in macrophage and immune function that we find in these mice (24). Interestingly, the role of p53 is not limited to tumor suppression, and this protein also possesses anti-atherogenic properties (42, 43). PLTP has been extensively characterized in the context of atherosclerosis and studies have shown that low levels of PLTP are associated with greater risk of atherosclerosis (44). Moreover, there is evidence that inhibition of ferroptosis alleviates atherosclerosis (45). Therefore, p53 may prevent atherosclerosis by inducing ferroptosis resistance through PLTP; this remains to be determined. Taken together, our findings provide evidence for a novel role for p53 and a p53 target gene in the regulation of ferroptosis. Whether PLTP also plays a role in tumor suppression by p53 remains a topic of investigation in the laboratory.

## Experimental procedures

### Mammalian cell culture, CRISPR knock-in cells

Human LCLs containing WT p53 (Catalog ID GM18870), the P47S variant (Catalog ID GM18871), and the Y107H variant (Catalog ID GM2634) were obtained from the Coriell Institute and grown in RPMI (Corning Cellgro) supplemented with 15% heat inactivated fetal bovine serum (HyClone, GE Healthcare Life Sciences) and 1% penicillin/streptomycin (Corning Cellgro). WT and P47S MEFs were generated as previously described (17) and maintained in complete growth medium. HepG2 cells were obtained from ATCC and maintained in complete growth medium. For the generation of HepG2 P47S cell lines using CRISPR/Cas9, the P47S point mutation was introduced by nucleofection with a synthetic gRNA/Cas9 ribonucleoprotein complex along with an ssODN. The gRNA recognition site for P47S is 5′-ACCATTGTTCAATATCGTCC*NGG*, with the PAM site italicized, and the ssODN has the following sequence with two phosphorothioate bonds at each end: 5′cttttcacccatctacagtcccccttgccgtcccaagcaatggatgatttgatgctgtccAGTgacgatattgaacaatggttcactgaagacccaggtccagatgaagctcccagaatg. Both synthetic gRNAs and ssODNs in ultramer format were purchased from Integrated DNA Technologies. The transfected pools of cells were analyzed by using Next Generation Sequencing for knock-in rate, and single-cell clones were obtained by sorting on a Sony sorter and screened by using Next Gen Sequencing. Positive clones were expanded, and all clones were confirmed to be negative for *mycoplasma* contamination and authenticated as HepG2 cells by STR profiling (GEIC, Washington University St Louis). P47S clones were confirmed to possess no other mutations by sequencing the TP53 cDNA in its entirety (Wistar Institute Genomics Facility). Complete growth medium is composed of Dulbecco’s modified Eagle’s medium (DMEM, Corning Cellgro, 4.5 g/l glucose) supplemented with 10% fetal bovine serum (HyClone, GE Healthcare Life Sciences) and 1% penicillin/streptomycin (Corning Cellgro). Cells were grown in a 5% CO_2_ humidified incubator at 37 °C. For low glucose/low serum experiments, cells were grown in DMEM containing 1 g/l glucose (Gibco 11885084) and 1% fetal bovine serum.

### Western blot, immunofluorescence, immunohistochemistry

Protein lysates were obtained from cell lines and 50 to 100 μg of protein was run on SDS-PAGE gels using 10% NuPAGE Bis-Tris precast gels (Life Technologies). Proteins were transferred onto polyvinylidene difluoride membranes (IPVH00010, pore size: 0.45 mm; Millipore Sigma) and blocked for 1 h in 5% milk. Primary antibodies include PLTP 1:1000 (Abcam ab18990 and Invitrogen MA5-42930) and GAPDH 1:10,000 (14C10, Cell Signaling, 2118). HRP-conjugated rabbit secondary antibody (Jackson Immunochemicals) was used at a 1:10,000 dilution. Blots were treated for 5 min with ECL (Amersham, RPN2232) and autoradiography was used to determine protein levels. For immunofluorescence, cells were pretreated overnight with oleic acid (Sigma, O3008), the DGAT2 inhibitor PF-06424439 (Sigma PZ0233), or vehicle (fatty acid–free bovine serum albumin [BSA]). Samples were then treated with 2 μM of Bodipy 493/503 (ThermoFisher D3922) for 15 min at 37 °C in the dark. The cells were mounted with media containing DAPI and images were captured using the Leica TSC SP5 microscope. For immunohistochemistry, liver tissues from 4- to 6-month-old male and female mice were fixed in formalin overnight at 4 °C. The following day, tissues were washed in 1× PBS and placed in 70% ethanol. Tissue embedding and sectioning was performed by the Wistar Institute Histotechnology Facility. Paraffin-embedded sections were deparaffinized in xylene (Fisher, X5-SK4), rehydrated in ethanol (100%-95%-85%-75%), and then placed in distilled water. Sample slides were steamed in 10 mM Citrate Buffer, pH 6, for antigen retrieval. Three percent hydrogen peroxide was used to quench endogenous peroxidase activity and slides were blocked for 1 h (Vector Laboratories, S-2012). The slides were incubated with the primary antibody PLTP (1:100, Santa Cruz sc-271596) overnight at 4 °C. The next day, slides were washed with PBS, incubated with HRP-conjugated secondary antibody for 30 min, and treated with DAB chromogen (D5637). Hematoxylin was used to perform a light counterstaining. The Nikon 80i upright microscope was used to image slides, at least four fields per section were imaged.

### RNA-seq, qRT-PCR

WT and P47S LCLs were treated with 5 μM nutlin for 30 min followed by the addition of 25 μM TBH for 0, 8, and 24 h. RNA was extracted using RNeasy kits (Qiagen) following the manufacturer’s protocol. The QuantSeq FWD library preparation kit (Lexogen) was used to generate 3′ mRNA-seq libraries from DNase I–treated RNA. The Agilent Tapestation and Agilent DNA 5000 Screentape were used to determine overall library size and real-time PCR (Kapa Biosystems) was used to quantitate the libraries. Libraries were pooled and the NextSeq 500 (Illumina) was used to carry out high-out-put single-read 75-base-pair next-generation sequencing. Bowtie2 (46) was used to align RNA-seq data against the human genome version hg38. Raw read counts for each gene were estimated using RSEM version 1.2.12 software (47). The significance of differential expression between samples was determined using DESeq2 (48). For qRT-PCR, RNA was extracted from cells using the RNeasy kits (Qiagen) following the manufacturer’s protocol. Equal amounts of the isolated RNA were used to generate cDNA using a high-capacity reverse transcription kit (Applied Biosciences, 4368814). The Brilliant III UltraFast SYBR Green qPCR mix kits (Agilent) were used to conduct qPCR on the Stratagene M × 3005P machine (Agilent). RNA expression levels were normalized to housekeeping gene 18S for human and cyclophilin A for mouse. Primer sequences are as follows: PLTP (human-F) 5′-TGATTGACTCCCCATTGAAGC-3′ and (human-R) 5′-CGTCCATAGTCATGCTGGACA-3′. Pltp (mouse-F) 5′-TTCCTCCTCAACCAGCAGATCT-3′ and (mouse-R) 5′-CAGGAGGGAGTTGAGCAACAC-3′.

### Silencing and overexpression studies

PLTP knockdown cell lines were generated by lentiviral infection, using the vector pLKO.1-puro–carrying shRNA sequence against human PLTP (shRNA1 [CTGATGCTTCAAATCACCAAT; TRCN0000150129], shRNA2 [CGAATCTATTCCAACCATTCT; TRCN0000148250]) and human TP53 (TCAGACCTATGGAAACTACTT, TRCN0000003754). Short hairpin constructs and packaging vectors were cotransfected in 293-FT cells to generate lentivirus. HepG2 cells were infected with lentivirus with 8 μg/ml polybrene, spun for 30 min at 2250 rpm, allowed to rest for 3.5 h followed by a media change. Puromycin was added the following day at 2 μg/ml, and gene knockdown was validated by qRT-PCR. Cells overexpressing PLTP were generated by transfecting the vector pcDNA3.1+/C-(K)DYK with and without PLTP ORF (NM_006227.3) obtained from GenScript. Transfections were performed with Lipofectamine 3000 (ThermoFisher) following the manufacturer’s protocol.

### Flow cytometry/Lipid peroxidation assay

Analysis of lipid peroxidation was performed using C11-BODIPY 581/591 (ThermoFisher D3861) and analysis of lipid droplet accumulation was performed using BODIPY 493/503 (ThermoFisher D3922). Cells were plated at a density of 3 × 10^5^ cells per well in 6-well plates. The following day, cells were treated with 0.5 μM of RSL3 for 3 h. Cells were collected, washed with PBS, and stained with 5 μM of C11-Bodipy for 30 min at 37 °C for lipid peroxidation analysis or 2 μM of Bodipy 493/503 for 15 min at 37 °C for lipid droplet analysis. Cells were then washed with PBS three times and analyzed using FACSCelesta (Becton Dickinson) flow cytometer. Dead cells were removed from analysis using FSC/SSC profiles, and cell doublets were eliminated by comparing forward scatter signal height *versus* forward scatter signal area. At least, 10,000 events in the analysis gate were obtained. Lipid peroxidation was determined for each sample by calculating the ratio of oxidized lipid signal intensity (510 nm) to unoxidized lipid signal intensity (590 nm).

### PLTP enzymatic activity assay

Cells were seeded at a density of 2 to 5 × 10^5^ cells/well in 6-well plates and grown overnight at 37 °C. The following day, cells were washed with PBS and incubated in NP-40 lysis buffer for 20 min at 4 °C. The protein lysates were diluted (1:500–1:5000) and used in enzymatic activity assays following the instructions in the PLTP Activity Assay Kit (Sigma Aldrich, MAK108) in 96-well opaque-bottom plates. The activity levels of the samples were measured using a Gen5 (Biotek) microplate reader.

### Viability assays

Cells were seeded at 2000 cells per well on a 96-well plate and were grown overnight at 37 °C. The following day, cells were treated with the following drugs at indicated concentrations for 72 h for IC_50_ assays: erastin (Cayman Chemicals), RSL3 (Apex Bio), TBH(Sigma), nutlin-3a (Calbiochem), cisplatin (Acros Organics), Etoposide (Sigma), Camptothecin (Cayman Chemicals), and doxorubicin (Cell Signaling). Because fetal bovine serum and culture media can contain significant levels of glutathione, pyruvate, and other agents that can influence sensitivity to ferroptosis (10), all ferroptosis sensitivity assays were performed in low serum/low glucose (2% fetal bovine serum, DMEM low glucose (Corning Cellgro, 1 g/l glucose)). For rescue assays, cells were pretreated with ferrostatin (Cayman Chemicals) or liproxstatin (Sigma) for 30 min prior to treatment with RSL3 for 24 h. For lipid droplet assays, cells were pretreated overnight with oleic acid (Sigma, O3008), the DGAT2 inhibitor PF-06424439 (Sigma, PZ0233), or vehicle (fatty-acid–free BSA), and viability was assayed using Trypan Blue (Corning, 25-900-CI) after 72 h. At assay endpoint, cells were treated with Alamar blue (Life Technologies Dal1025) for 3 h at 37 °C and absorbance was read using a SynergyHT plate reader (BioTek). GraphPad Prism software was used to perform the data analysis.

### Statistical analysis

All experiments were performed in three biological replicates done in triplicate unless otherwise stated, and the two-tailed unpaired student *t* test was performed to determine significance. All *in vitro* data are reported as the mean ± SD unless stated otherwise. Statistical analyses were performed using GraphPad Prism, *p*-values are as follows: (∗) *p*-value < 0.05, (∗∗) *p*-value < 0.01, (∗∗∗) *p*-value < 0.001, (∗∗∗∗) *p*-value < 0.0001.

## Data availability

The RNA Sequencing data described in this article is deposited in the GEO database (GSE202265).

## Supporting information

This article contains supporting information.

## Conflict of interest

The authors declare that they have no conflicts of interest with the contents of this article.
