# Supplementary figures and images for: PLTP is a p53 target gene with roles in cancer growth suppression and ferroptosis
Source: J Biol Chem. 2022 Oct 26;298(12):102637. doi: 10.1016/j.jbc.2022.102637 (PMC9709240; doi:10.1016/j.jbc.2022.102637)

Supplemental Figure 1

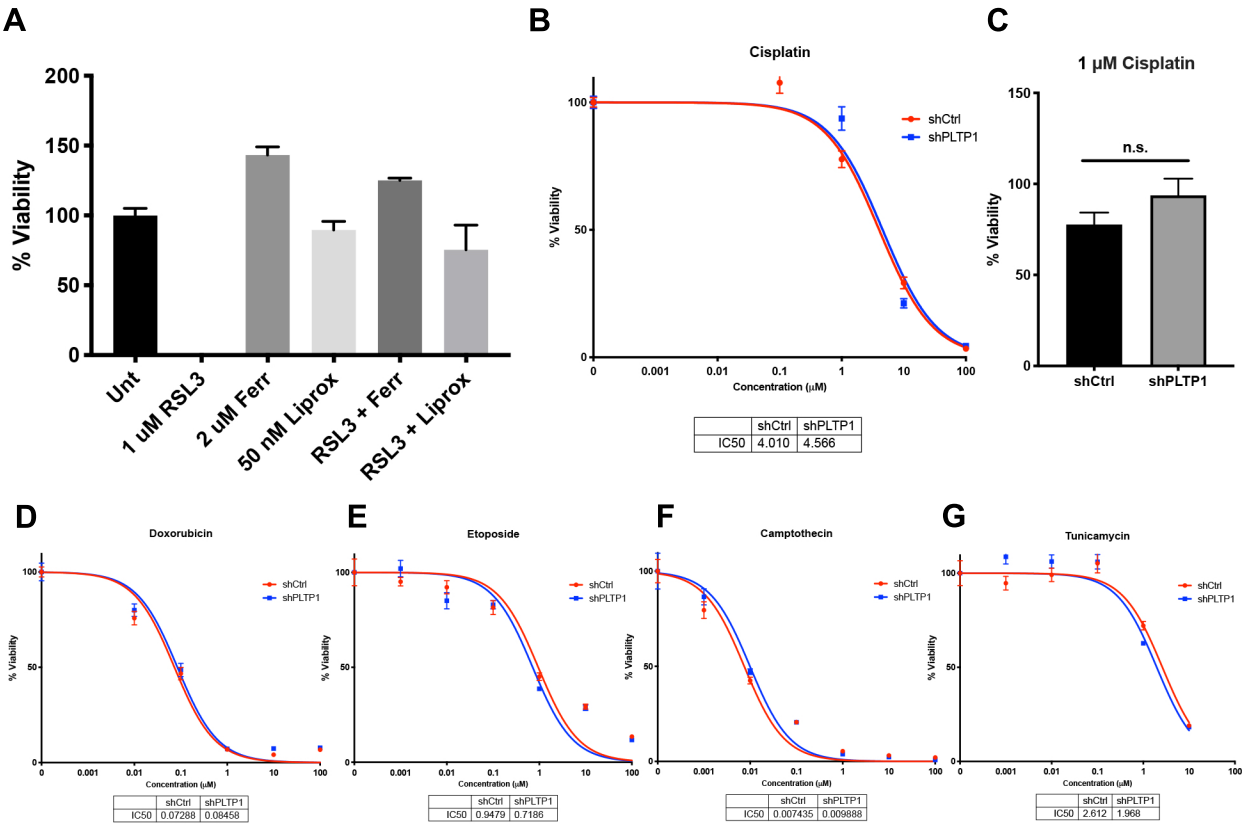

Supplement: Supplemental Figure 1 [file mmc1.pdf]
