# Supplementary material for: PLTP is a p53 target gene with roles in cancer growth suppression and ferroptosis
Source: J Biol Chem. 2022 Oct 26;298(12):102637. doi: 10.1016/j.jbc.2022.102637 (PMC9709240; doi:10.1016/j.jbc.2022.102637)
Supplement: Supplemental Figure 1 Legend [file mmc2.docx]

**Supplemental Figure 1.** **PLTP silencing does not affect sensitivity to other forms of cell death.**

(A) Viability analysis of PLTP knockdown cells subjected to indicated treatments. Cells were pretreated with either 2 µM ferrostatin or 50 nM liproxstatin for 30 minutes, followed by treatment with 1 µM RSL3 for 24 hours. Viability normalized to untreated cells, error bars indicate standard deviation, n = 4.

(B) Viability analysis of short hairpin control or PLTP knockdown cells treated with indicated doses of cisplatin for 72 hours. Error bars represent standard error of mean, n = 4.

(C) Viability analysis of short hairpin control or PLTP knockdown cells treated 1 µM cisplatin for 72 hours (single point from b). Error bars indicate standard error of the mean; p-value not significant.

(D-G) Viability analysis of short hairpin control or PLTP knockdown cells treated with indicated doses of (d) doxorubicin, (e) etoposide, (f) camptothecin, or (g) tunicamycin for 72 hours. Error bars represent standard error of mean, n = 4. No significant differences observed.
